# GLI pathogenesis-related 1 functions as a tumor-suppressor in lung cancer

**DOI:** 10.1186/s12943-016-0508-4

**Published:** 2016-03-18

**Authors:** Xiumei Sheng, Nathan Bowen, Zhengxin Wang

**Affiliations:** School of Medicine, Jiangsu University, Zhenjiang, Jiangsu 212013 China; The Center for Cancer Research and Therapeutic Development, Department of Biological Sciences, Clark Atlanta University, 223 James P. Brawley Drive, S.W., Atlanta, Georgia 30314 USA

**Keywords:** Lung, Lung cancer, Tumor suppressor, GLIPR1, ErbB3, PRMT5, WDR77

## Abstract

**Background:**

GLI pathogenesis-related 1 (GLIPR1) was originally identified in glioblastomas and its expression was also found to be down-regulated in prostate cancer. Functional studies revealed both growth suppression and proapoptotic activities for GLIPR1 in multiple cancer cell lines. GLIPR1’s role in lung cancer has not been investigated. Protein arginine methyltransferase 5 (PRMT5) is a protein arginine methyltransferase and forms a stoichiometric complex with the WD repeat domain 77 (WDR77) protein. Both PRMT5 and WDR77 are essential for growth of lung epithelial and cancer cells. But additional gene products that interact genetically or biochemichally with PRMT5 and WDR77 in the control of lung cancer cell growth are not characterized.

**Methods:**

DNA microarray and immunostaining were used to detect GLIPR1 expression during lung development and lung tumorigenesis. *GLIPR1* expression was also analyzed in the TCGA lung cancer cohort. The consequence of GLIPR1 on growth of lung cancer cells in the tissue culture and lung tumor xenografts in the nude mice was observed.

**Results:**

We found that GLIPR1 expression is negatively associated with PRMT5/WDR77. GLIPR1 is absent in growing epithelial cells at the early stages of mouse lung development and highly expressed in the adult lung. Expression of GLIPR1 was down-regulated during lung tumorigenesis and its expression suppressed growth of lung cancer cells in the tissue culture and lung tumor xenografts in mice. GLIPR1 regulates lung cancer growth through the V-Erb-B avian erythroblastic leukemia viral oncogene homolog 3 (ErbB3).

**Conclusions:**

This study reveals a novel pathway that PRMT5/WDR77 regulates GLIPR1 expression to control lung cancer cell growth and GLIPR1 as a potential therapeutic agent for lung cancer.

**Electronic supplementary material:**

The online version of this article (doi:10.1186/s12943-016-0508-4) contains supplementary material, which is available to authorized users.

## Background

GLI pathogenesis-related 1 (GLIPR1) is similar to both the pathogenesis-related protein superfamily and the cysteine-rich secretory protein family [1–4]. It is also referred as the related to testes-specific, vespid, and pathogenesis protein 1 (RTVP-1), originally identified in human glioblastomas [2, 3]. GLIPR1 is highly expressed in gliomas and astrocytic brain malignancies, and its expression level is correlated with the degree of malignancy of astrocytic tumors [2, 3, 5]. GLIPR1 overexpression increased cell proliferation, survival, invasion, migration and anchorage-independent growth of glioma cells [5]. GLIPR1 was variably expressed in metastatic melanoma and elevated GLIPR1 levels were correlated with increased invasive potential [6]. Elevated GLIPR1 expression was also detected in Wilms tumors [7]. In contrast, GLIPR1 expression was down-regulated in prostate cancer and functions as a tumor-suppressor gene in prostate cancer [8, 9]. Overexpression of GLIPR1 induced apoptosis [10] and/or mitotic catastrophe (MC) in prostate cancer cells [11]. Adenoviral vector-mediated *GLIPR1* therapy in an immunocompetent orthotopic prostate mouse model showed significantly reduced tumor-associated angiogenesis [12]. A novel *GLIPR1*-modified tumor cell vaccine also showed significant antitumor activity in a mouse model of recurrent prostate cancer [13]. Furthermore, a phase I clinical trial of neoadjuvant intraprostatic injection of *GLIPR1* delivered by adenoviral vector for localized and intermediate and high-risk prostate cancer before radical prostatectomy showed antitumor activity and favorable modulation of blood-based biomarkers of immune stimulation [14].

V-Erb-B avian erythroblastic leukemia viral oncogene homologs (ErbBs) belong to the family of tyrosine kinase receptors, which containing four members (ErbB1/EGFR, ErbB2/Her2, ErbB3/Her3, and ErbB4) [15, 16]. Insufficient ErbB signaling in humans is associated with the development of neurodegenerative diseases, while excessive ErbB signaling is associated with the development of a wide variety of types of solid tumors [17, 18]. These cell surface receptors are comprised of a composite extracellular domain which contains a well defined ligand-binding site, a single pass transmembrane domain, and an intracellular domain with tyrosine kinase activity [17, 19]. Ligand binding induces homo or heterodimerization between ErbB receptors, leading to activation of their tyrosine kinase activity, and activation of multiple downstream pathways [20, 21]. It was reported that ERBB3 played a major role in division, survival, motility, migration, and invasiveness of lung cancer cells [22, 23] and high ERBB3 expression was also associated with poor prognosis in lung cancer patients [24–26].

Protein arginine methyltransferase 5 (PRMT5) is a type II protein arginine methyltransferase that catalyzes the symmetrical dimethylation of arginine residues within target proteins and has been implicated in diverse cellular and biological processes [27]. PRMT5 forms a stoichiometric complex with the WD repeat domain 77 (WDR77/MEP50/WD45/p44) in various cells [28–30]. PRMT5 and WDR77 proteins in the cytoplasm are required for proliferation of prostate epithelial and prostate cancer cells [31–36]. In contrast, in the nucleus, they function with the androgen receptor to drive prostate epithelial cell differentiation and function [33, 34, 37]. More recently, we found that WDR77 is highly expressed in the lung at the early development stage when cells are rapidly proliferating and its expression is diminished in adult lung when cells are fully differentiated [31]. Loss of WDR77 expression led to growth arrest of lung epithelial cells at the G1 cell cycle phase. More important, PRMT5 and WDR77 were re-activated in lung cancers and the small hairpin RNA (shRNA)-mediated silencing of PRMT5 or WDR77 expression strongly inhibited growth of lung cancer cells in the tissue culture and abolished growth of lung tumor xenografts in the nude mouse [31, 32]. These results reveal a novel role of PRMT5 and WDR77 in growth of lung epithelial cells as well as lung cancers.

In searching for genes that mediate PRMT5 and WDR77 functions in lung cancer cells, we performed DNA microarray analysis (GSE56757) with lung adenocarcinoma A549 cells expressing WDR77 or PRMT5 shRNA [32, 31] and found that the loss of WDR77 or PRMT5 expression significantly up-regulated GLIPR1 expression. GLIPR1 expression was down-regulated during lung tumorigenesis and re-expression of GLIPR1 inhibited proliferation of lung cancer cells and growth of lung tumor xenografts. This study identifies GLIPR1 as a tumor suppressor for lung cancers.

## Results and discussion

### GLIPR1 expression was suppressed by WDR77 in lung cancer cells

**Fig. 1:**
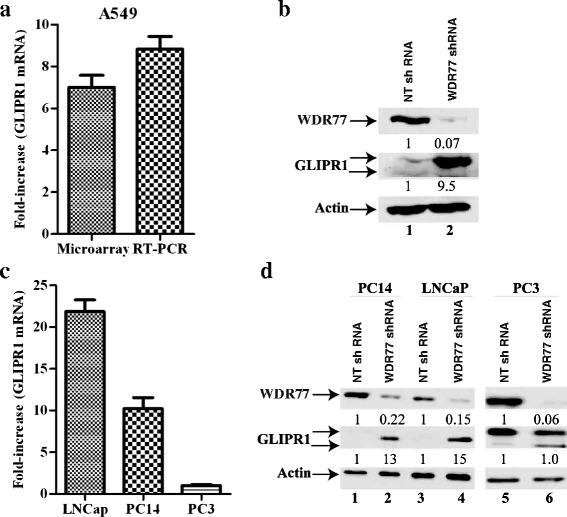
GLIPR1 expression was suppressed by WDR77 in lung and prostate cancer cells. **a** DNA microarray and RT-RCR analysis of GLIPR1 mRNA expression in A549 cells. Fold-increase = GLIPR1 mRNA signals of cells expressing WDR77 shRNA/GLIPR1 signals of cells expressing NT shRNA. **b** Western Blot analysis of whole-cell lysates derived from A549 cells expressing NT shRNA (lane 1) or WDR77 shRNA (lane 2) with anti-WDR77, anti-GLIPR1 or anti-actin antibodies as indicated. **c** RT-RCR analysis of GLIPR1 mRNA expression in LNCaP, PC14, or PC3 cells. Fold-increase = GLIPR1 mRNA signals of cells expressing WDR77 shRNA/GLIPR1 signals of cells expressing NT shRNA. **d** Western Blot analysis of whole-cell lysates derived from PC14, LNCaP, or PC3 cells expressing NT shRNA (lane 1, 3, 5) or WDR77 shRNA (lane 2, 4, 6) with anti-WDR77, -CLIPR1, or -actin antibody as indicated

Silencing expression of WDR77 or PRMT5 dramatically inhibited proliferation of lung (A549 and PC14) and prostate (PC3 and LNCaP) cancer cells [32, 36]. To investigate potential molecular mechanisms through which WDR77/PRMT5 functions, we performed DNA microarray expression profiling and found that *GLIPR1* gene expression was up-regulated by 7-fold in WDR77-silenced A549 cells (Fig. 1a) and 11-fold in PRMT5-silenced A549 cells (GSE56757), which was confirmed by an RT-PCR analysis (Fig. 1a). GLIPR1 protein levels were also significantly (9.4-fold) higher in WDR77-silenced A549 cells comparing to the control A549 cells (Fig. 1b, lane 2 versus lane 1). The anti-GLIPR1 antibody detected two protein bands, the upper one represents the N-glycosylated form of GLIPR1 [46]. These results suggest that WDR77 suppresses GLIPR1 expression in lung cancer A549 cells. We further tested GLIPR1 expression in the other WDR77-silenced lung (PC14) and prostate (LNCaP and PC3) cancer cells. Both mRNA (Fig. 1c) and protein (Fig. 1d) levels of GLIPR1 were higher in WDR77 shRNA-expressing LNCaP and PC14 cells compared with non-target (NT) shRNA-expressing LNCaP and PC14 cells (Fig. 1d, lane 2 versus lane 1, lane 4 versus lane 3). However, silencing WDR77 expression did not enhance GLIPR1 expression in PC3 cells (Fig. 1c; Fig. 1d, lane 6 versus lane 5). We noticed that GLIPR1 protein levels are low in A549, PC14 and LNCaP cells but high in PC3 and glioma U87 cells (Fig. 1b, lane 1, Fig. 1d, lanes 1, 3, 5; Additional file 1: Figure S1). Consistent with these observations, we also found that GLIPR1 mRNA levels in PC3 cells are also much higher than that in A549, PC14 and LNCaP cells. These results indicate that GLIPR1 expression is inversely associated with WDR77 in A549, PC14 and LNCaP cells and such association is absent in PC3 cells. Furthermore, the DNA microarray analysis (GDS1688) indicates that GLIPR1 expression in all of the tested lung cancer cell lines except PC-6 is lower than that in A549 cells (Additional file 2: Figure S2), which is associated with high expression of WDR77 in these cell lines (Additional file 3: Figure S3).

**Fig. 2:**
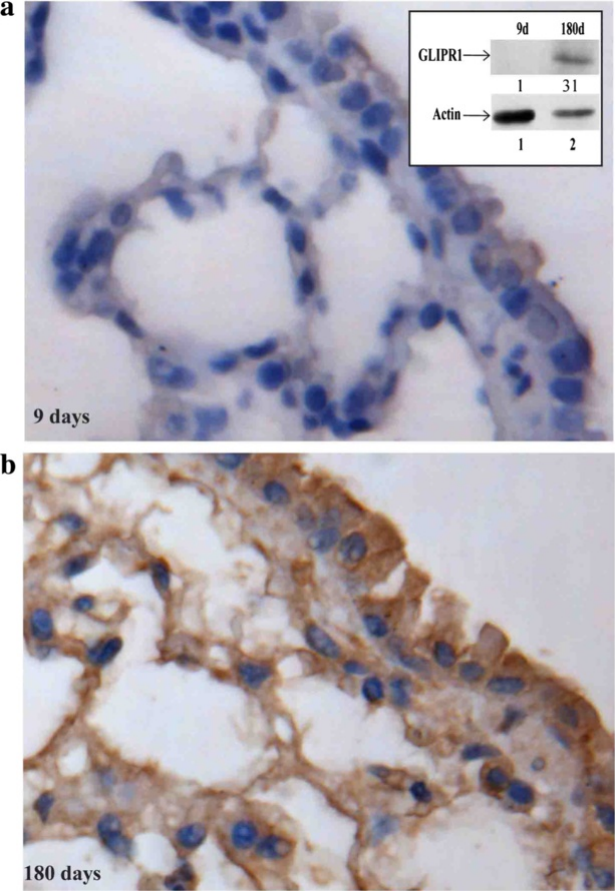
GLIPR1 expression is up-regulated during mouse lung development. Lung tissues obtained from mice at the age of 9 (**a**) or 180 (**b**) days were immunostained using the anti-GLIPR1 antibody. GLIPR1-expressing cells are stained in brown. The insert shows Western blot analysis of protein extracts made from the lungs of mice at the age of 9 (lane 1) or 180 (lane 2) days using the anti-GLIPR1 antibody

Immunostaining of mouse lung tissues with the anti-GLIPR1 antibody was performed to detect GLIPR1 expression. At the age of 9 days after birth, GLIPR1 is very low in the mouse lung epithelial and alveolar cells (Fig. 2a). At the age of 180 days, high GLIPR1 levels in the cytoplasm of both epithelial and alveolar cells were detected (Fig. 2b). Western blot analysis confirmed this observation (Fig. 2a, insert). In our previous study, we reported that WDR77 was highly expressed in the lung of mouse at the age of 1–14 days, while absent in the lung of mouse at the age of 120–390 days. Thus, GLIPR1 expression is inversely correlated with WDR77 expression during the lung development. Consistent with this conclusion, the DNA microarray analysis (GDS3950) demonstrated that GLIPR1 expression is also inversely correlated with WDR77 expression during the mouse lung development from the embryonic day 12 to the postnatal day 30 (Additional file 4: Figure S4).

**Fig. 3:**
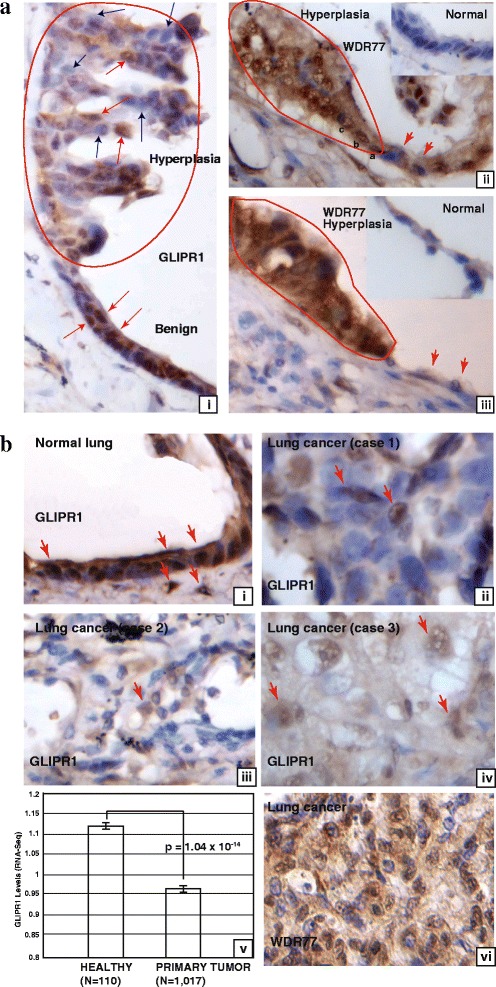
GLIPR1 expression is down-regulated during lung tumorigenesis. Immunostaining of GLIPR1 or WDR77 in normal lung tissues (**a**-ii, insert panels; **b**-i), lung hyperplasia (**a**, surrounded by *red lines*), and lung cancer (**b**). GLIPR1 or WDR77-expressing cells are stained in *brown*. (**b**-v), GLIPR1 levels in healthy and primary lung tumor tissues. Average normalized RSEM values from TCGA (lung) RNA-Seq gene expression data set (https://genome-cancer.ucsc.edu/download/public/xena/TCGA/TCGA.LUNG.sampleMap/HiSeqV2)

We then examined GLIPR1 expression in lung cancer samples derived from 35 patients. GLIPR1 was highly expressed in benign (Fig. 3ai, indicated by red arrows) and normal (Fig. 3bi) lung epithelial cells. In the lung hyperplasia region (Fig. 3ai, circled by red line), some cells lost GLIPR1 expression (indicated by blue arrows) but the others remain GLIPR1-staining positive with the decreased GLIPR1 protein levels (indicated by red arrows). In contrast, WDR77 expression is absent in normal lung epithelial (Fig. 3aii, insert) and alveolar (Fig. 3aiii, insert) cells but robust and ubiquitous in lung hyperplasia derived from lung epithelial cells (Fig. 3aii, circled by the red line) or from alveolar cells (Fig. 3aiii, circled by the red line). The expression of WDR77 is also ubiquitous in lung cancers (Fig. 3bvi). In contrast, GLIPR1 expression is variable in all 35 lung cancer samples (Three cases of lung cancers are shown in Fig. 3bii/iii/iv.) with some lung cancer cells expressing GLIPR1 at the decreased protein levels (Fig. 3bii/iii/iv, Some are indicated by red arrows but others not.). Consistent with this observation, GLIPR1 expression is also decreased in lung cancers [cohort: the Cancer Genome Atlas (TGCA) lung cancer, *n* = 1,127] (Fig. 3bv). It was also shown that expression of WDR77 and GLIPR1 has the tendency towards mutual exclusivity in 230 lung cancers [38]. Thus, GLIPR1 expression is down-regulated and inversely correlated with WDR77 expression during lung tumorigenesis. Consistent with this conclusion, gene expression microarray analysis (GDS4794) demonstrated that *GLIPR1* expression is down-regulated in 23 small cell lung cancers (SCLC) (Additional file 5: Figure S5).

### WDR77 regulates GLIPR1 expression through the TGFβ signaling

**Fig. 4:**
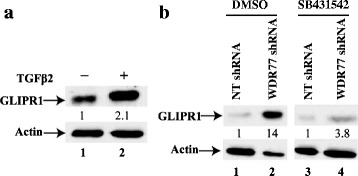
WDR77 regulates GLIPR1 expression through the TGFβ signaling. **a** Western Blot analysis of whole-cell lysates derived from A549 cells grown in the absence or presence of the recombinant human TGFβ2 (10 ng/ml, Biolegend) (lane 2) for 24 h. **b** Western Blot analysis of whole cell extracts made from A549 cells expressing NT shRNA treated with DMSO (lane 1) or SB431542 (10 μM) (lane 2) and A549 cells expressing WDR77 shRNA treated with DMSO (lane 3) or SB431542 (lane 4) for 24 h

In our previous study, we found that loss of WDR77 expression activated the TGFβ signaling pathway through up-regulation of TGFβ2 and TGFβII expression [39]. To test whether the TGFβ signaling mediates the WDR77-regulated GLIPR1 expression, we first treated A549 cells with the recombinant human TGFβ2. TGFβ2 enhanced GLIPR1 expression by 2.1-fold (Fig. 4a, lane 2 versus lane 1). We then treated A549 cells that express WDR77 or NT shRNA with SB431542, a TGFβ receptor inhibitor. Silencing WDR77 induced GLIPR1 protein expression by 14-fold (Fig. 4b, lane 2 versus lane 1), which was greatly suppressed by SB431542 (lane 4 versus lane 3). These results suggest that the TGFβ signaling pathway participates in GLIPR1 up regulation upon WDR77 knockdown. Our current efforts are focused on how WDR77 suppresses the TGFβ signaling which in turn regulates *GLIPR1* gene expression.

GLIPR1 was identified as a TP53 target gene [40]. Silencing WDR77 expression slightly (2-fold) decreased TP53 protein level in LNCaP cells (Additional file 6: Figure S6A). We constructed a TP53 luciferase reporter (pGL3-4xPBE-E4-luc), which contains four copies of the TP53 DNA-binding sequence (PBE) fused to the E4 core promoter and luciferase gene. This reporter was transiently transfected into LNCaP cells that expressed NT or WDR77 shRNA and the promoter activity was determined by measuring the luciferase activity. Silencing WDR77 also slightly decreased the p53-reporter activity (Additional file 6: Figure S6B). Thus, WDR77 regulates *GLIPR1* gene expression not through p53.

### GLIPR1 inhibits cell proliferation and arrests cell cycle at G1 and G2 phases

**Fig. 5:**
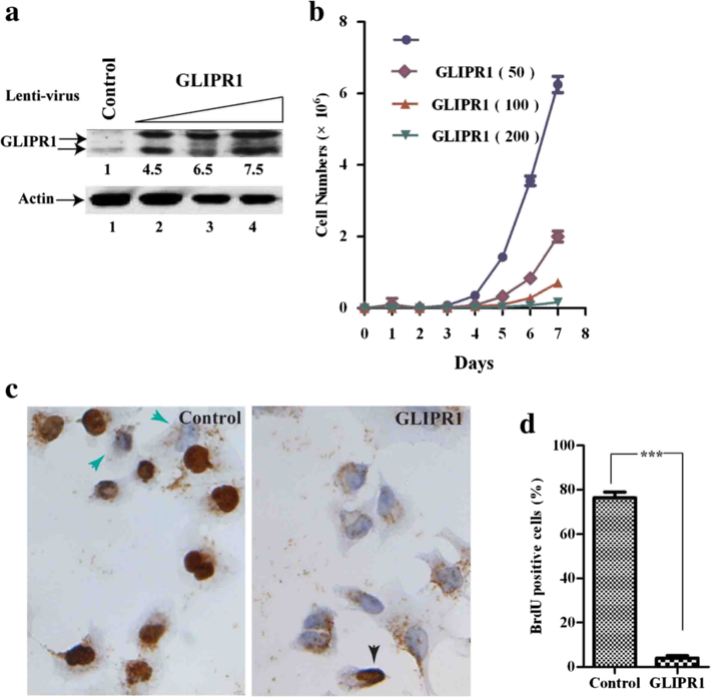
GLIPR1 inhibits cell proliferation. **a** Western blot analysis of whole-cell lysates derived from A549 cells infected with the control lentivirus (lane 1) or with variable amounts (50, 100, or 200 μl) of lentivirus expressing GLIPR1 (lanes 2–4). **b** Growth curves of control A549 cells or A549 cells expressing different levels of GLIPR1. **c** A549 cells infected with control lentivirus or GLIPR1-expressing lentivirus were allowed to grow in the presence of BrdU and immunostained with the anti-BrdU antibody (*brown*). The green arrow (*left-hand panel*) indicates the BrdU-negative staining cell and the black arrows (*right-hand panel*) indicate the BrdU-positive staining cells. **d** Percentage of BrdU-positive cells in A549 cells infected with control lentivirus or GLIPR1-expressing lentivirus. The results represent the means of three independent experiments ± the standard error of the mean (SEM). *, *P*<0.05; **, *P*<0.01; ***, *P*<0.001

We showed previously that silencing WDR77 expression significantly decreased proliferation of prostate and lung cancer cells [39, 41]. We then investigated whether GLIPR1 influences growth of lung cancer cells. A549 cells were infected with lentivirus expressing GLIPR1 protein. GLIPR1 protein levels were increased (up to 7.5-fold) in A549 cells in a lentiviral dosage-dependent manner (Fig. 5a, lanes 2–4). GLIPR1 over expression resulted in a significant inhibition of cell growth and the cell growth rate is inversely correlated with GLIPR1 protein levels (Fig. 5b).

We then performed a BrdU incorporation assay to measure the proliferation rate of control and GLIPR1-expressing A549 cells. About 75 % control cells are stained positively by the anti-BrdU antibody (Fig. 5c, left-hand panel, in brown; Fig. 5d), indicating the majority of control cells are proliferative. In contrast, only about 5 % of GLIPR1-expressing cells stained positively for BrdU. (Fig. 5c, right-hand panel, indicated by the black arrow; Fig. 5d). Thus, GLIPR1 expression is negatively correlated with cellular proliferation. Flow cytometry analysis revealed no significant difference in the apoptosis rate (sub G cell population) between GLIPR1-expressing and the control A549 cells (Additional file 7: Figure S7), indicating that GLIPR1 expression did not induce apoptosis in lung cancer cells. However, the proportion of the GLIPR1-expressing cells in the G1- and G2-phases of the cell cycle was significantly higher than that in control cells (Additional file 7: Figure S7). Conversely, the proportion of GLIPR1 expressing cells found in S-phase was lower than that in control A549 cells. Thus, GLIPR1 inhibits cellular proliferation by arresting cell cycle at G1 and G2 phases.

### GLIPR1 down regulates ErbB2/3 expression

**Fig. 6:**
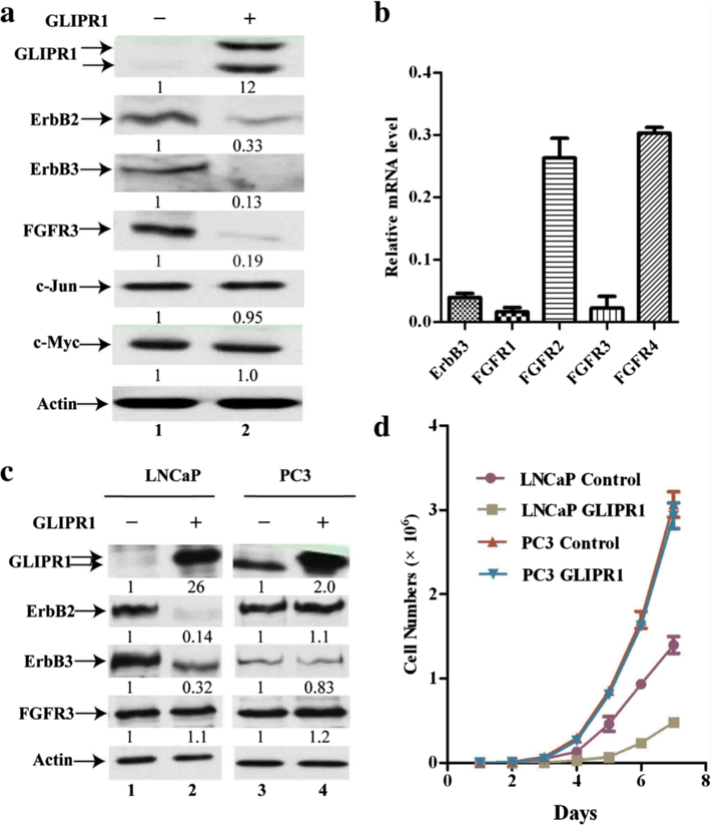
GLIPR1 down regulates expression of ERBB2/3 genes. **a** Western blot analysis of whole-cell lysates derived from control A549 cells (lane 1) or GLIPR1-expressing A549 cells (lane 2) with antibodies as indicated. **b** RT-RCR analysis of ERBB3, FGFR1, FGFR2, FGFR3, and FGFR4 expression. Relative mRNA level = mRNA in A549 cells expressing GLIPR1/mRNA in the control A549 cells. **c** Western blot analysis of whole-cell lysates derived from control LNCaP cells (lane 1), GLIPR1-expressing LNCaP cells (lane 2), control PC3 cells (lane 3), or GLIPR1-expressing PC3 cells (lane 4) with antibodies as indicated. **d** Growth curves of control LNCaP cells, GLIPR1-expressing LNCaP cells, control PC3 cells or GLIPR1-expressing PC3 cells

Our previously reported gene expression microarray experiments demonstrated that silencing WDR77 or PRMT5 down-regulated ERBB2/3 and FGFR3 gene expression (GSE56757) [33]. Western blotting analysis showed that GLIPR1 expression also decreased expression of FGFR3, ERBB2, and ERBB3 proteins in A549 cells (Fig. 6a, lane 2 versus lane 1). RT-PCR analysis indicated that this regulation was at the mRNA level (Fig. 6b). Besides, the expression of FGFR1/2/4 was down-regulated by GLIPR1 as well, which may also contribute to GLIPR1’s suppressor function (Fig. 6b). It was reported that GLIPR1 overexpression led to activation of the c-Jun-NH2 kinase pathway in a TSU-Pr1-derived cell line [42] and stimulated CK1α-mediated β-catenin and c-Myc destruction in multiple prostate cancer cell lines [10]. However, GLIPR1 expression did not alter c-Jun and c-Myc protein levels in A549 cells (Fig. 6a, lane 2 versus lane 1). Similarly, we also expressed GLIPR1 in prostate cancer LNCaP and PC3 cells (Fig. 6c) and compared with control cells, GLIPR1 protein levels were increased by 26.6- and 2-fold in GLIPR1-expressing LNCaP (lanes 1 and 2) and PC3 (lanes 3 and 4) cells, respectively (Fig. 6c). GLIPR1 expression decreased the protein levels of ERBB2 and ERBB3, but did not alter FGFR3 protein levels in LNCaP cells (Fig. 6c, lane 2 versus lane 1). In contrast, GLIPR1 expression did not alter levels of any of these three proteins in PC3 cells (Fig. 6c, lane 4 versus lane 3). GLIPR1 expression also inhibited growth of LNCaP but not PC3 cells (Fig. 6d).

**Fig. 7:**
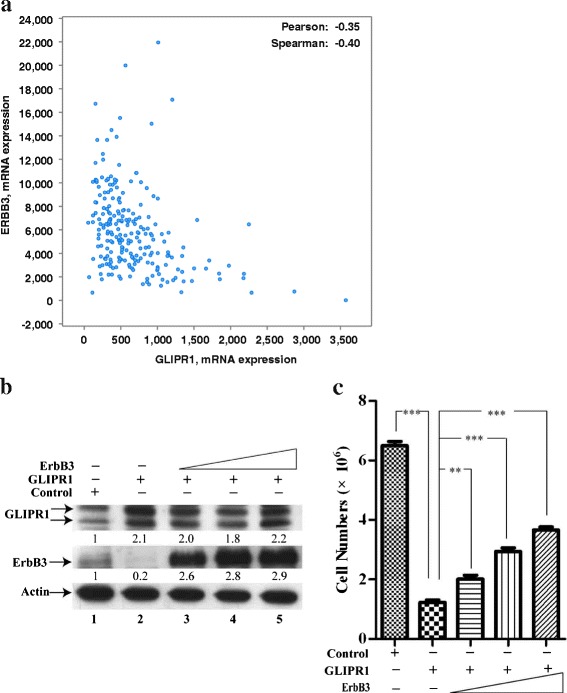
**a** ErbB3 expression is inversely correlated with GLIPR1 expression in lung cancers. mRNA co-expression of GLIPR1 versus ErbB3 in 230 lung cancer samples. Data was obtained from the Cancer Genome Atlas (TCGA, lung cancer, 2014) and analyzed using cBioPortal (www.cbioportal.org). **b**, **c** Ectopic expression of ErbB3 partially restores cell growth inhibition by GLIPR1. **b** Western blot analysis of whole-cell lysates derived from control A549 cells (lane 1) or A549 cells expressing GLIPR1 or GLIPR1 plus ERBB3. **c** Growth of A549 cells expressing GLIPR1 or GLIPR1 plus ERBB3. **, *P*<0.01; ***, *P*<0.001.

ERBB3 expression is also inversely correlated with GLIPR1 expression in 230 lung cancer samples (Fig. 7a) [38]. Ectopic expression of ERBB3 (Fig. 7b) partially restored the cell growth inhibition induced by GLIPR1 expression in A549 cells (Fig. 7c). Thus, these results indicate that ErbB3 may participate in cell growth inhibition mediated by GLIPR1. However, ERBB3 expression failed to fully restore the cell growth inhibition induced by GLIPR1 expression. ERBB2 lacks ligand-binding ability, whereas ERBB3 is unique in that it does not have any intrinsic kinase activity [43, 44]. ERBB2 and ERBB3 form the heterodimer and perform a central role in maintenance and malignancy of lung cancers and other cancers [24, 26, 45–47]. Combined blockade of ERBB2 and ERBB3 inhibited PI3K/Akt activity more effectively than each inhibitor alone [45, 47]. It may be possible that ERBB2 and ERBB3 should be co-expressed in order to fully restored growth inhibition induced by GLIPR1. It is possible that other signal pathways such as FGFR also mediate growth-suppression function of GLIPR1.

### GLIPR1 suppressed lung tumor growth in an orthotopic mouse model

**Fig. 8:**
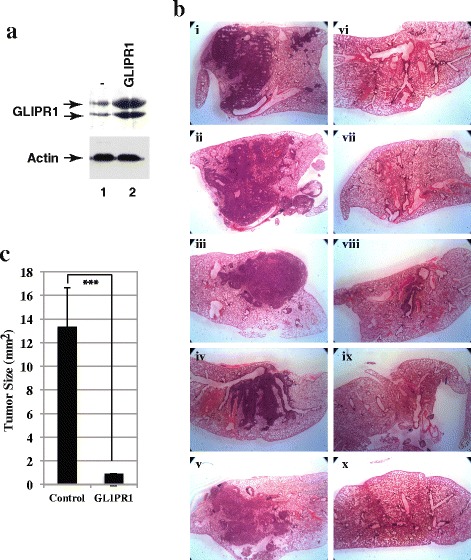
GLIPR1 inhibited growth of lung tumor xenografts. **a** Western blot analysis of whole-cell lysates derived from control A549 cells (lane 1) or GLIPR1-expressing A549 cells (lane 2) with anti-GLIPR1 (*top*) or anti-actin (*bottom*) antibody. **b** Lungs derived from mice injected with control A549 cells (panels i-v) or A549 cells expressing GLIPR1 (panels vi-x). **c** Mean size of tumors in mouse lungs injected with the control A549 cells or A549 cells expressing GLIPR1. Vertical bars indicate SEM. ***, *P*<0.001

To determine the effects of GLIPR1 on lung cancer, we observed the growth of orthotopic tumors in mice formed from the control A549 cells or A549 cells expressing GLIPR1 (Fig. 8a). Large, macroscopically visible tumors were found in the lungs of the mice injected with the control A549 cells (Fig. 8b, panels i-v). However, only a small tumor was observed in the lung of one mouse injected with GLIPR1-expressing A549 cells (Fig. 8b, pane viii). No tumors were detected in the lungs of the other 4 mice injected with GLIPR1-expressing A549 cells (Fig. 8b, panels vi, vii, ix, x). The average tumor size (tumor area mean) in the mice injected with the control A549 cells was 15-fold higher than that of the lung tumors in mice injected with GLIPR1-expressing A549 cells (Fig. 8c). These results indicate that GLIPR1 functions as a tumor suppressor in lung tumors.

### GLIPR1 performs dual functions in PC3 cells

**Fig. 9:**
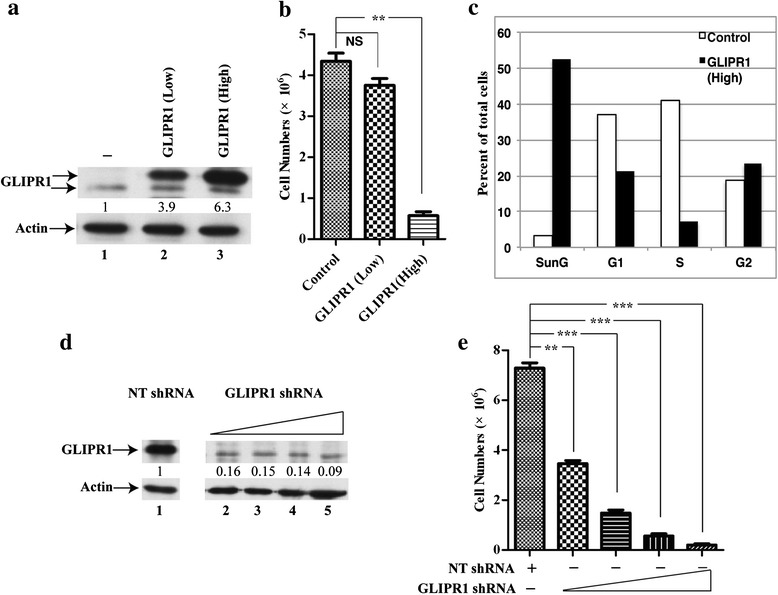
Dual functions of GLIPR1 in PC3 cells. **a** Western blot analysis of whole-cell lysates derived from control PC3 cells (lane 1), PC3 cell infected with low (lane 2) or high (lane 3) amounts of GLIPR1-expressing lentivirus. **b** Cell numbers 6 days after infected with control lentivirus or GLIPR1-expressing lentivirus. **c** The cell-cycle distribution in control or high GLIPR1-expressing lentivirus infected PC3 cells by using flow cytometric analysis. **d** Western blot analysis of whole-cell lysates derived from PC3 cells expressing NT shRNA (lane 1) or GLIPR1 shRNA (lane 2–5) with anti-GLIPR1 or anti-actin antibody. **e** Cell numbers 6 days after infected with NT shRNA-expressing or GLIPR1 shRNA expressing lentivirus. **, *P*<0.01; ***, *P*<0.001

GLIPR1 protein levels in PC3 are much higher than that in A549, PC14, and LNCaP cells (Additional file 1: Figure S1) and 2-fold increase in GLIPR1 protein levels did not affect growth of PC3 cells (Fig. 6c, d). However, previous studies showed that GLIPR1 overexpression induced apoptosis in PC3 cells [40]. By carefully examining GLIPR1 protein levels, we found that the adenovirus-mediated expression in the previous study [40] yielded much higher GLIPR1 protein levels than the lentivirus-mediated GLIPR1 expression used here. We constructed another GLIPR1-expressing lentivirus by inserting three deoxynucleotides (ACC) immediately before the translation initiation codon (ATG) of GLIPR1 cDNA, which significantly enhanced the translation efficiency [48, 49]. Indeed, GLIPR1 protein levels in PC3 cell could be increased up to 6.3-fold (Fig. 9a, lane 3 versus lane 1). High levels of GLIPR1 significantly inhibited PC3 cell growth (Fig. 9b). Flow cytometry cell cycle analysis confirmed that high GLIPR1 expression indeed induced apoptosis in about 50 % cells (Fig. 9c), which is similar to the published results [40]. In addition, high GLIPR1 expression also significantly decreased the cell population found in S phase (Fig. 9c).

It was reported that GLIPR1 was highly expressed in glioma and functioned as an oncogene [2, 5]. We then tested whether the endogenous GLIPR1 protein performs any function in PC3 cells. PC3 cells were infected with lentivirus expressing the NT or GLIPR1 shRNA. GLIPR1 shRNA significantly decreased GLIPR1 protein expression in PC3 (Fig. 9d, lanes 2–5 versus lane 1). Silencing GLIPR1 expression inhibited PC3 cell growth in a dosage dependent manner (Fig. 9e). This result is consistent with the published observations in glioma cells [2, 5]. Therefore, GLIPR1 expression is required for PC3 cell growth, while overexpression of GLIPR1 at much higher levels inhibits PC3 cell growth by inducing cell apoptosis. These results are consistent with the observed role of GLIPR1 in glioma and Wilms tumors [20, 22]. A possible explanation for the conflicting cancer-related functions of GLIPR1 is that it may form complexes with various factors that have distinct functions. The GLIPR1 protein deleted of the N-glycosylation site [48] inhibited cell growth as well as the wild-type GLIPR1 (Data not shown), indicating that the N-glycosylation of GLIPR1 does not affect its function to suppress lung cancer cell growth.

## Conclusions

Silencing WDR77 or PRMT5 expression in lung cancer A549 cells induced expression of *GLPIR1* revealed by DNA microarray (GSE56757) and RT-PCR analyses. Such regulation also exists in other lung (PC14) and prostate (LNCaP) cancer cells. *GLIPR1* expression is inversely correlated with WDR77 during mouse lung development. WDR77 and PRMT5 are ubiquitously re-expressed in lung hyperplasia and cancer [31, 32] and conversely, GLIPR1 expression is significantly down-regulated during lung tumorigenesis. Thus, these results suggest the negative regulation of the *GLIPR1* gene by WDR77.

Our mechanistic studies show that GLIPR1 inhibits expression of ERBB2/3 in A549 and LNCaP cells. Moreover, ERBB3 expression is inversely correlated with GLIPR1 in 230 lung cancers [38]. Ectopic expression of ERBB3 partially restores growth inhibition induced by GLIPR1, suggesting that GLIPR1 inhibits lung cancer cell growth through suppressing ERBB3.

Although GLIPR1 has been reported to possess tumor suppressor activities in prostate cancer cells [40, 50], its function in lung cancer have not yet been reported. We found that GLIPR1 inhibited expression of ERBB2/3 and proliferation of both lung cancer (A549, PC14) and prostate cancer (LNCaP) cells. Thus, GLIPR1 may serve as a novel therapeutic agent for lung cancer by using either gene or protein delivery methods. Such methods are currently under phase I clinical trial of prostate cancer treatment [13, 14].

## Methods

### RNA interference and gene expression profiling

The WDR77 and non-target (NT) small hairpin RNAs (shRNAs) were described previously. The GLIPR1 shRNA target sequence (5’-GGA CUA UGA CUU CAAGAC Udtdt-3’) was designed [51] and cloned into the lentiviral gene transfer vector pLVTHM. The DNA construct was sequenced to determine the proper insertion. The lentivirus production and cell infection were performed as describe. The infected cells were then submitted for growth assay and harvested for Western blot and real-time PCR analyses. A gene expression profiling analysis (GSE56757) was performed on A549 cells expressing WDR77, PRMT5, or NT shRNA. The Cancer Genome (TCGA) data of GLIPR1 expression in lung samples were retrieved from https://genome-cancer.ucsc.edu/download/public/xena/TCGA/TCGA.LUNG.sampleMap/HiSeqV2 and analyzed for significant expression differences using RSEM [52].

### Real-time PCR

Total RNAs were isolated from cultured cells using TRIzol reagent and reverse transcribed into cDNA using the Reaction Ready First Strand cDNA Synthesis Kit (SuperArray Bioscience Corp.). The cDNA products were PCR-amplified with the RT^2^ Real-Time SYBR Green PCR master mix (SuperArray Bioscience Corp.) and the gene-specific primer sets (Additional file 8: Table S1) for human GLIPR1, FGFR1, FGFR2, FGFR3, FGFR4, ErbB3, and β-actin genes (Sigma) using a SmartCycler II (Cepheid; 40 cycles of 30 s at 94 °C, 30 s at 55 °C and 30 s at 72 °C). The SmartCycler software program (version 2.0C) was used to process and quantify raw data. The 2^*-ΔΔCT*^ method was used to relatively quantify target gene expression as described previously [53].

### Western blot analysis

Protein extracts made from the cultured cells were subjected to 10 % sodium dodecyl sulfate-polyacrylamide gel electrophoresis and then transferred to Immobilon-P membranes (Millipore). The blots were then probed for 2 h with primary antibodies at dilutions of 1:1,000 (anti-WDR77), 1:1,000 (anti-FGFR3), 1:1,000 (anti-c-Myc, Santa Cruz Biotechnology), 1:1,000 (anti-c-Jun, Santa Cruz Biotechnology), or 1:5,000 (anti-β-actin, Sigma-Aldrich) or overnight with primary antibody at dilutions of 1:1,000 (anti-ErbB3), 1:1,000 (anti-ErbB2), or 1:1,000 (anti-GLIPR1). The blots were then incubated with a horseradish peroxide-conjugated secondary antibody for 1.5 h. Immunoreactive proteins were detected using an enhanced chemiluminescence detection system (GE Healthcare) per the manufacturer’s instructions. Protein concentrations were determined using the Bradford protein assay (Bio-Rad). The protein bands were scanned using a densitometer, and the relative intensities were quantified using the ImageJ software program (ImageJ64, National Institutes of Health).

### Lung samples and immunohistochemical staining

Lung tumor samples were obtained from patients with lung cancer who underwent surgery at Tangdu Hospital (Xi’an, China), and the study protocol was approved by its institutional review board. The confidential information of patients cannot be traced from lung tumor samples. BALB/c mice were purchased from the National Cancer Institute and maintained in the Animal Facility of Morehouse Medical School. Mouse lung tissues preparation was performed as described previously. Mice were handled in accordance with the guidelines published in the National Institutes of Health Guide for the Care and Use of Laboratory Animals. The Morehouse Medical School’s Institutional Animal Care and Use Committee approved all the experimental procedures used for mice. Antigen retrieval and immunostaining with anti-GLIPR1 (Abnova, 1:200) or -WDR77 (1:1,000) antibody were performed as described [32]. Immunostaining without the primary antibody served as a negative control.

### Cell culture, cell growth assay, and the treatment with TGFβ2

A549 and PC14 cells were cultured in minimum essential medium (Cellgro) with 10 % (v/v) fetal bovine serum (FBS) (HyClone), 2 % vitamins, 1 % L-glutamine, 1 % non-essential amino acids, and 1 % sodium pyruvate. PC3 and LNCaP cells were cultured in RPMI 1640 medium (Cellgro) with 10 % FBS. For the growth assay, cells were plated on 24-well plates (2, 000 cells/well) and counted every day for 7 days. For bromodeoxyuridine (BrdU) (BD Biosciences) incorporation assay, cells (50–70 % confluence) were plated on a Chamber slide (BD falcon) and cultured in the presence of 10 μM BrdU for 4 h. The BrdU-positive cells were detected by immunostaining with the monoclonal anti-BrdU antibody (BD Biosciences).

For the TGFβ2 treatment, A549 cells were seeded in 6-well plates in the growth medium. The fresh medium containing 10 ng/ml recombinant human TGFβ2 (Biolegend) was added 24 h later. Cells were cultured for 24 h in a CO_2_ incubator and were harvested for Western blot analysis. A549 cells were first transfected with lentivirus expressing non-target (NT)-shRNA or WDR77 shRNA and 3 days post infection, the fresh medium containing either 0.1 % DMSO or 10 μM SB431542 (Reagents Direct) was then added. Cells were harvested for Western blot analysis 24 h later. Each experiment was performed in triplicate.

### Flow cytometry cell cycle analysis

Cells (50–60 % confluency) were harvested, washed with phosphate-buffered saline (PBS), and fixed in 70 % ethanol at 4 °C overnight. Cells were collected and stained with propidium iodide (PI). The cell-cycle distributions were determined by flow cytometry analysis (BD Accuri^TM^ C6 Flow Cyometer).

### Ectopic expression of GLIPR1

Human GLIPR1 cDNA clone (HsCD00441029) in the lentiviral expression vector pLX304 was purchased from DNASU (www.dnasu.org/DNASU/Home.do). To increase GLIPR1 expression, three deoxynucleotides (ACC) were inserted into the GLIPR1 cDNA just before the initiation codon (ATG). Lentivirus was prepared as described previously. A549 cells (4 ×10^4^) were plated in 6-well plates and transduced with GLIPR1-expressing lentivirus or control lentivirus. After 48 h, cells were replated in 100-mm dish, and 2 days later GLIPR1 expression was confirmed by Western Blotting analysis.

### Lung orthotopic tumors

Six-week-old nude mice were purchased from the National Cancer Institute and maintained in a barred animal facility. Cells injection into mouse lung was performed as described previously. Mice were killed 21 days after tumor cell injection. The lungs of the mice were then removed, fixed with formaldehyde, and embedded in paraffin. Paraffin-embedded lung tissue sections (4 μm) were stained with H&E and evaluated for tumors. Mice were handled in accordance with the guidelines published in the National Institutes of Health Guide for the Care and Use of Laboratory Animals. The Morehouse Medical School Institutional Animal Care and Use Committee approved all the experimental procedures used for mice. The tumor areas in lungs were quantified using the ImageJ software program.

### Statistical analysis

Data are presented as the means of three independent experiments ± the standard error of the mean (SEM). A 2-tailed unpaired Student *t*-test was used to determine whether differences between control and experiment samples were statistically significant. *P* values less than 0.05 were considered statistically significant.

## Additional files

Additional file 1: Figure S1. GLIPR1 expression in A549, PC14, LNCaP, PC3 and U87 cells. Western blot analysis of whole-cell lysates derived from A549 (lane 1), PC14 (lane2), LNCaP (lane 3), PC3 (lane 4), and U87 (lane 5) cells with anti-GLIPR1 or -actin antibody. (PDF 308 kb) Additional file 2: Figure S2. The expression profile of *GLIPR1* in various lung cancer cell lines. The results were obtained from expression profiling (GDS1688) of a set of 29 lung cancer cell lines consisting of ten non-small cell adenocarcinoma, ten small cell cancer, and nine squamous cell cancer lines. Value: the RMA normalized expression value. Rank: the position of the *GLIPR1* gene across 22,337 genes on the DNA chip based on the expression level (from low to high). (PDF 403 kb) Additional file 3: Figure S3. The expression profile of *WDR77* in various lung cancer cell lines. The results were obtained from expression profiling (GDS1688) of a set of 29 lung cancer cell lines consisting of ten non-small cell adenocarcinoma, ten small cell cancer, and nine squamous cell cancer lines. Value: the RMA normalized expression value. Rank: the position of the *WDR77* gene across 22,337 genes on the DNA chip based on the expression level (from low to high). (PDF 428 kb) Additional file 4: Figure S4 Supplementary Results were obtained from expression profiling (GDS3950) of lung from the mouse over a time course beginning at embryonic day 12 and continuing into adulthood, encompassing all recognized stages of lung development. (PDF 289 kb) Additional file 5: Figure S5. The expression profile of *GLIPR1* in small cell lung cancers. The results were obtained from expression profiling (GDS4794) of 23 clinical small cell lung cancer (SCLC) samples from patients undergoing pulmonary resection and the normal lung tissue sample. (PDF 352 kb) Additional file 6: Figure S6. WDR77 regulates GLIPR1 expression not through the p53 signaling. a Western blot analysis of whole-cell lysates derived from LNCaP cells expressing NT shRNA (lane 1) or WDR77 shRNA (lane 2) with anti-WDR77, −p53, or -actin. b Silencing WDR77 expression decreased the activity of the p53 reporter. LNCaP cells expressing NT or WDR77 shRNA were transfected with 150 ng of the reporter plasmid pGL3-4 × PBE-E4-luc. The transfected cells were allowed to grow for 48 h and then harvested for the luciferase assay (Promega). The values represent the mean ± SD (*n* = 3). (PDF 431 kb) Additional file 7: Figure S7. Cell-cycle distribution in A549 cells infected with control lentivirus or GLIPR1-expressing lentivirus by using flow cytometric analysis. (PDF 611 kb) Additional file 8: Table S1. List of primers used for RT-PCR analysis. (PDF 40 kb)

## Abbreviations

BrbB: V-Erb-B avian erythroblastic leukemia viral oncogene homolog
BrdU: bromodeoxyuridine
FBS: fetal bovine serum
FGFR: fibroblast growth factor receptor
GLIPR1: GLI pathogenesis-related 1
NT shRNA: non-target shRNA
PBS: phosphate-buffered saline
PI: propidium iodide
PI3K: phosphatidylinositol-4,5-bisphosphate 3-kinase
PRMT5: protein arginine methyltransferase 5
RT-PCR: reverse transcription polymerase chain reaction
SEM: the standard error of the mean
shRNA: small hairpin RNA
TGFβ: transforming growth factor beta
WDR77: WD repeat-containing protein 77
