# Supplementary figures and images for: GLI pathogenesis-related 1 functions as a tumor-suppressor in lung cancer
Source: Mol Cancer. 2016 Mar 18;15:25. doi: 10.1186/s12943-016-0508-4 (PMC4797332; doi:10.1186/s12943-016-0508-4)

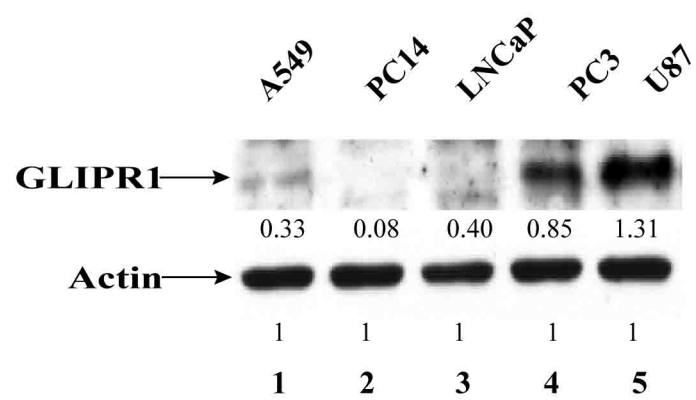

Supplement: Additional file 1: Figure S1. — GLIPR1 expression in A549, PC14, LNCaP, PC3 and U87 cells. Western blot analysis of whole-cell lysates derived from A549 (lane 1), PC14 (lane2), LNCaP (lane 3), PC3 (lane 4), and U87 (lane 5) cells with anti-GLIPR1 or -actin antibody. (PDF 308 kb) [file 12943_2016_508_MOESM1_ESM.pdf]

GDS3950 / 1422844\_a\_at / Wdr77

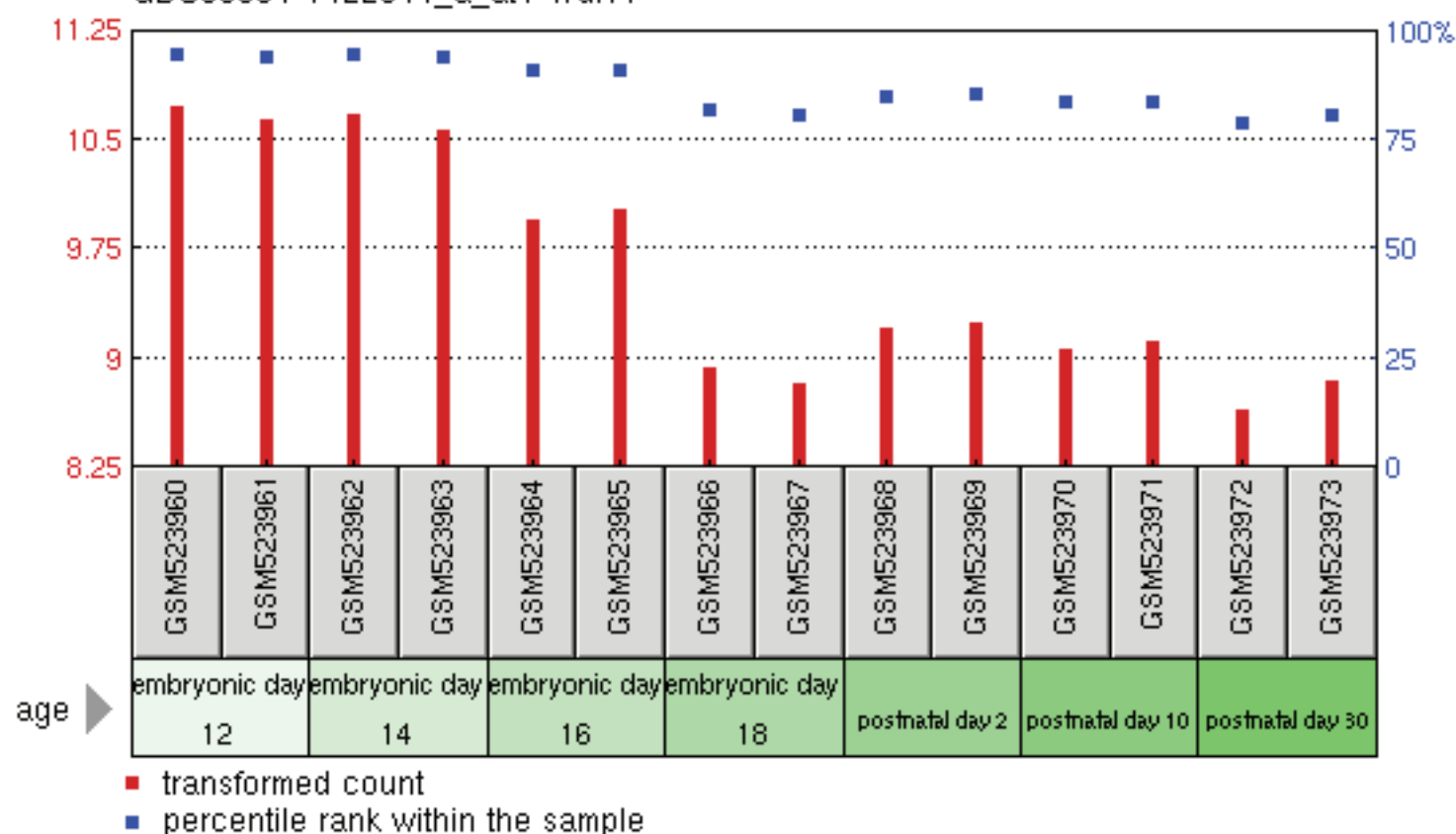

GDS3950 / 1424927\_at / Glipr1

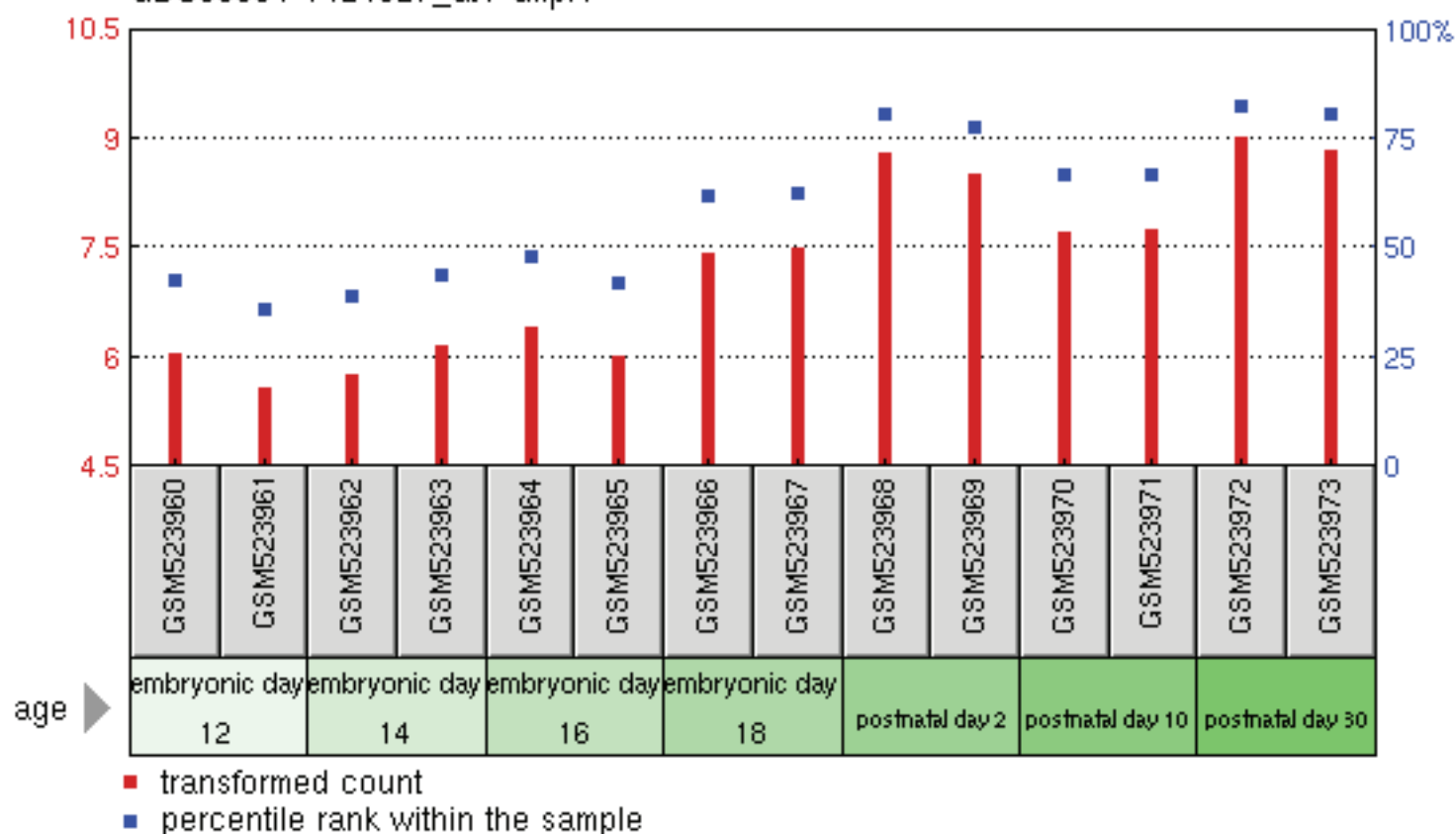

Supplement: Additional file 4: Figure S4 — Supplementary Results were obtained from expression profiling (GDS3950) of lung from the mouse over a time course beginning at embryonic day 12 and continuing into adulthood, encompassing all recognized stages of lung development. (PDF 289 kb) [file 12943_2016_508_MOESM4_ESM.pdf]

GDS 4794  
GLIPR1 expression in small cell lung cancers (SCLC)

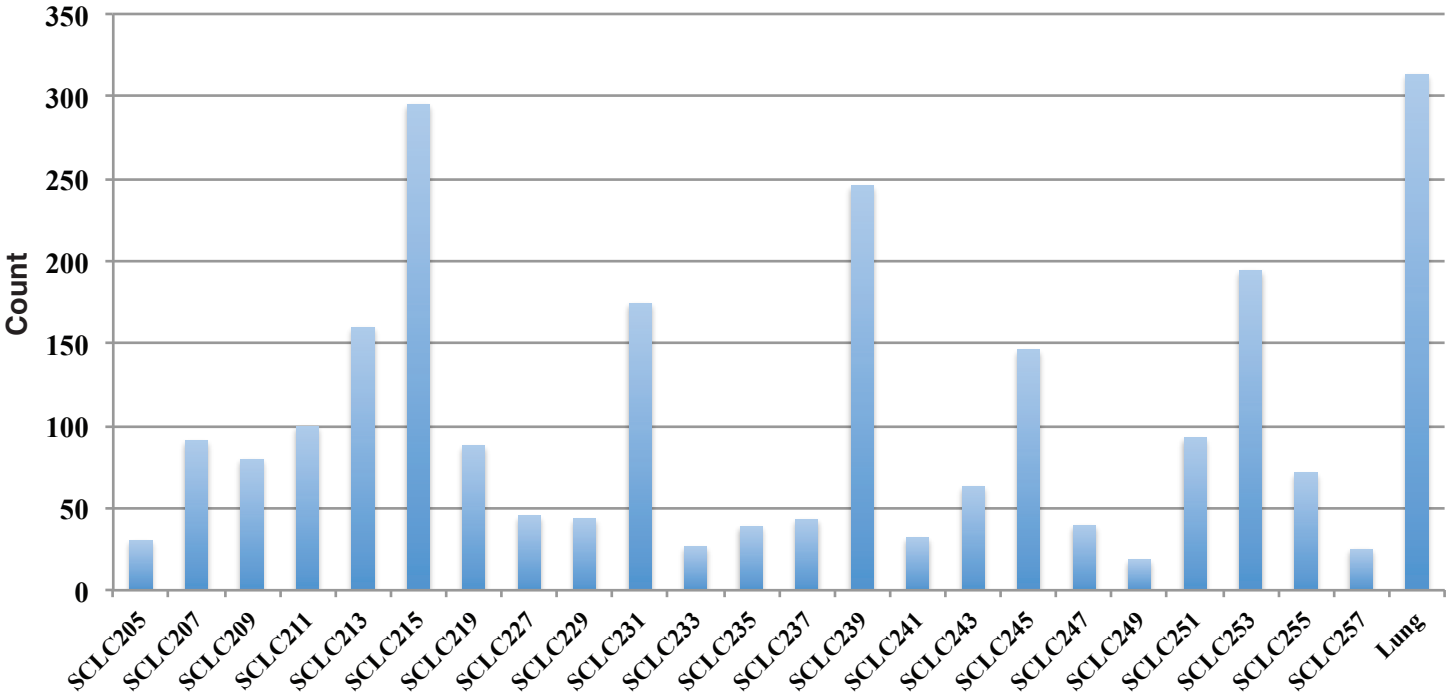

Supplement: Additional file 5: Figure S5. — The expression profile of GLIPR1 in small cell lung cancers. The results were obtained from expression profiling (GDS4794) of 23 clinical small cell lung cancer (SCLC) samples from patients undergoing pulmonary resection and the normal lung tissue sample. (PDF 352 kb) [file 12943_2016_508_MOESM5_ESM.pdf]

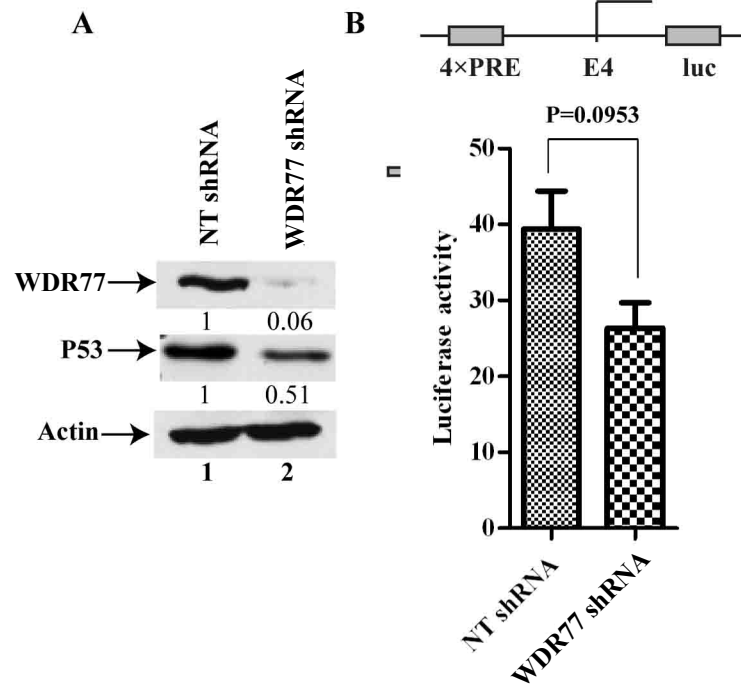

Supplement: Additional file 6: Figure S6. — WDR77 regulates GLIPR1 expression not through the p53 signaling. a Western blot analysis of whole-cell lysates derived from LNCaP cells expressing NT shRNA (lane 1) or WDR77 shRNA (lane 2) with anti-WDR77, −p53, or -actin. b Silencing WDR77 expression decreased the activity of the p53 reporter. LNCaP cells expressing NT or WDR77 shRNA were transfected with 150 ng of the reporter plasmid pGL3-4 × PBE-E4-luc. The transfected cells were allowed to grow for 48 h and then harvested for the luciferase assay (Promega). The values represent the mean ± SD (n = 3). (PDF 431 kb) [file 12943_2016_508_MOESM6_ESM.pdf]

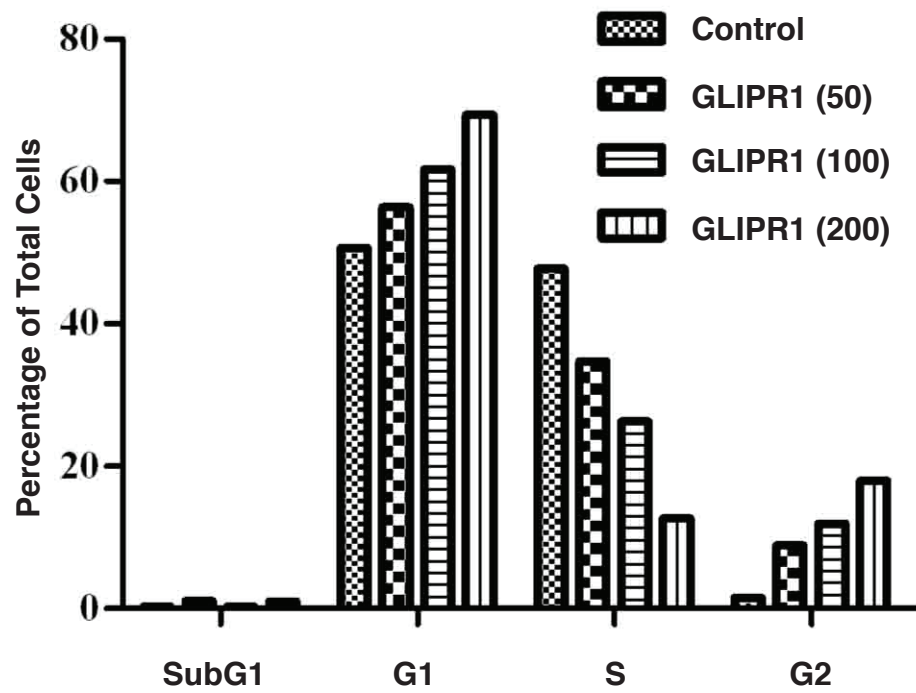

Supplement: Additional file 7: Figure S7. — Cell-cycle distribution in A549 cells infected with control lentivirus or GLIPR1-expressing lentivirus by using flow cytometric analysis. (PDF 611 kb) [file 12943_2016_508_MOESM7_ESM.pdf]
