# Supplementary material for: GLI pathogenesis-related 1 functions as a tumor-suppressor in lung cancer
Source: Mol Cancer. 2016 Mar 18;15:25. doi: 10.1186/s12943-016-0508-4 (PMC4797332; doi:10.1186/s12943-016-0508-4)
Supplement: Additional file 2: Figure S2. — The expression profile of GLIPR1 in various lung cancer cell lines. The results were obtained from expression profiling (GDS1688) of a set of 29 lung cancer cell lines consisting of ten non-small cell adenocarcinoma, ten small cell cancer, and nine squamous cell cancer lines. Value: the RMA normalized expression value. Rank: the position of the GLIPR1 gene across 22,337 genes on the DNA chip based on the expression level (from low to high). (PDF 403 kb) [file 12943_2016_508_MOESM2_ESM.pdf]

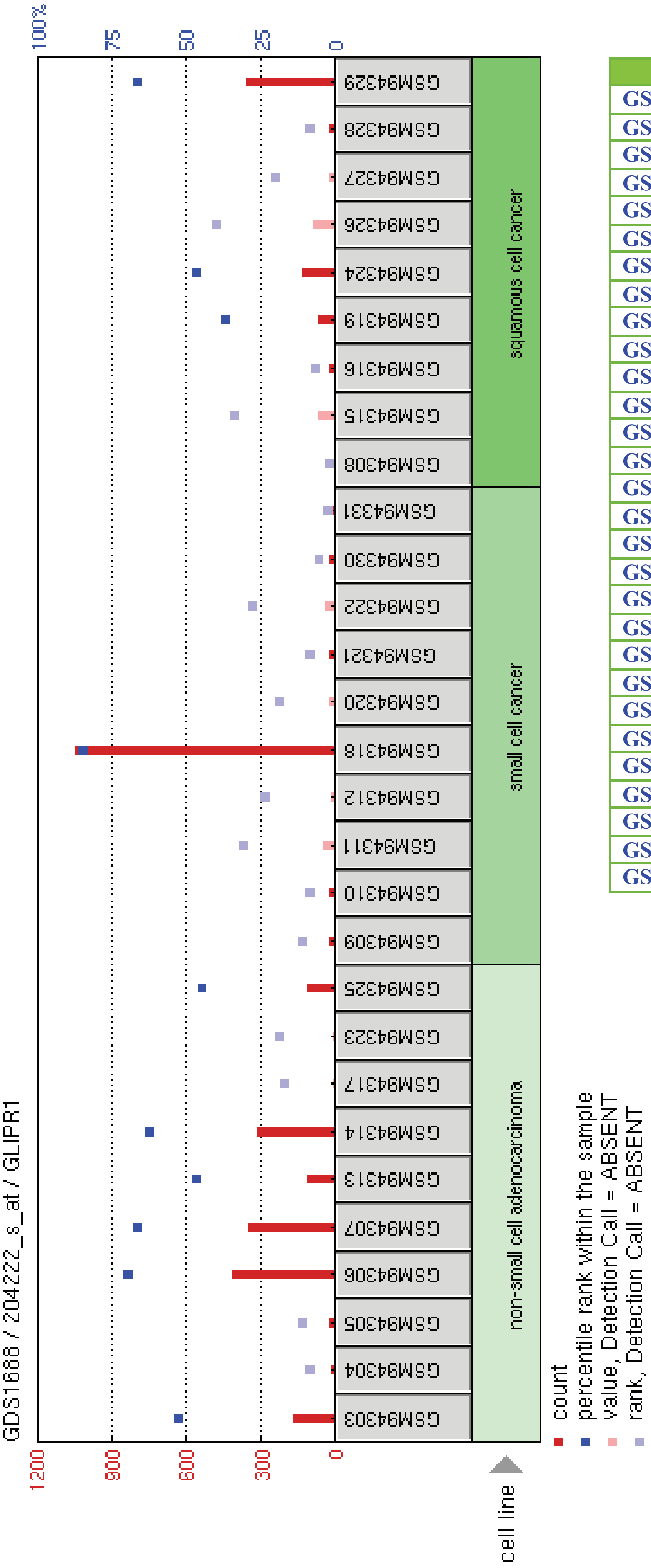

Sample	Title	Value	Rank
GSM94303	PC9	173.6	53
GSM94304	PC7	6.8	9
GSM94305	PC14	8	11
GSM94306	A549	422	70
GSM94307	LU65	358.2	67
GSM94313	RERF LC-KJ	122	47
GSM94314	RERF LC-MS	324.4	63
GSM94317	PC-3	17	17
GSM94323	ABC-1	21.3	19
GSM94325	LC2/ad	122	45
GSM94309	H69	8.7	11
GSM94310	N231	8	9
GSM94311	LU135	52.9	31
GSM94312	SBC3	25.6	24
GSM94318	PC-6	1053.6	85
GSM94320	Lu130	34.3	19
GSM94321	Lu139	8.1	9
GSM94322	Lu165	44.3	28
GSM94330	MS-1	7.4	6
GSM94331	SBC-5	6.4	3
GSM94308	LK2	4.1	2
GSM94315	RERF-LC-AI	76.6	34
GSM94316	PC-1	10.6	7
GSM94319	PC-10	76.6	37
GSM94324	EBC-1	138.2	47
GSM94326	LC1/sq	95.4	40
GSM94327	LC-1F	30.2	20
GSM94328	SQ-5	12.4	9
GSM94329	QG-56	364.4	67
