# Supplementary material for: GLI pathogenesis-related 1 functions as a tumor-suppressor in lung cancer
Source: Mol Cancer. 2016 Mar 18;15:25. doi: 10.1186/s12943-016-0508-4 (PMC4797332; doi:10.1186/s12943-016-0508-4)
Supplement: Additional file 3: Figure S3. — The expression profile of WDR77 in various lung cancer cell lines. The results were obtained from expression profiling (GDS1688) of a set of 29 lung cancer cell lines consisting of ten non-small cell adenocarcinoma, ten small cell cancer, and nine squamous cell cancer lines. Value: the RMA normalized expression value. Rank: the position of the WDR77 gene across 22,337 genes on the DNA chip based on the expression level (from low to high). (PDF 428 kb) [file 12943_2016_508_MOESM3_ESM.pdf]

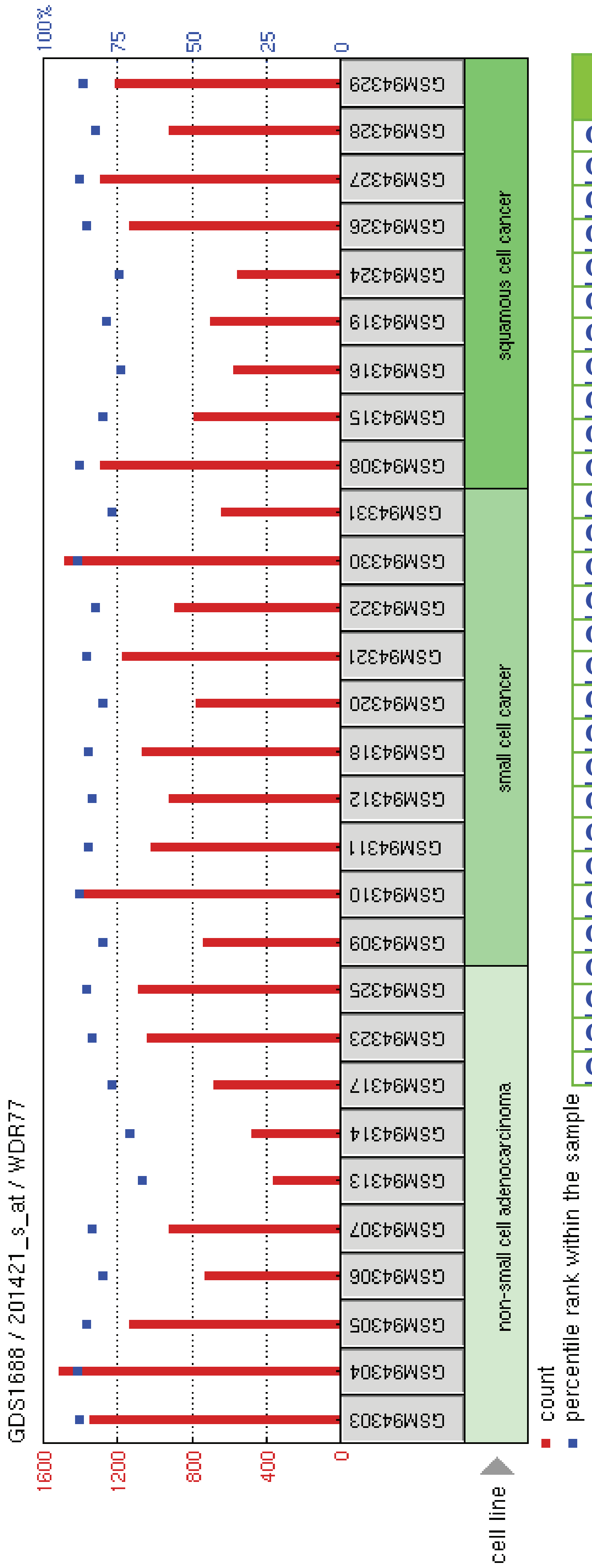

Sample	Title	Value	Rank
GSM94303	PC9	1353.8	88
GSM94304	PC7	1520.5	89
GSM94305	PC14	1138.6	86
GSM94306	A549	736.5	80
GSM94307	LU65	929.2	84
GSM94313	RERF LC-KJ	369.4	67
GSM94314	RERF LC-MS	487.4	71
GSM94317	PC-3	690	77
GSM94323	ABC-1	1049.3	84
GSM94325	LC2/ad	1096.6	86
GSM94309	H69	750.8	80
GSM94310	N231	1389	88
GSM94311	LU135	1024.2	85
GSM94312	SBC3	927.5	84
GSM94318	PC-6	1078.5	85
GSM94320	Lu130	788.7	80
GSM94321	Lu139	1183	86
GSM94322	Lu165	904.1	83
GSM94330	MS-1	1488.6	89
GSM94331	SBC-5	648.8	77
GSM94308	LK2	1291.9	88
GSM94315	RERF-LC-AI	793.6	80
GSM94316	PC-1	586.5	74
GSM94319	PC-10	711.7	79
GSM94324	EBC-1	560.8	75
GSM94326	LC1/sq	1146.5	86
GSM94327	LC-1F	1298.7	88
GSM94328	SQ-5	925.7	83
GSM94329	QG-56	1214.7	87
