# Supplementary material for: GLI pathogenesis-related 1 functions as a tumor-suppressor in lung cancer
Source: Mol Cancer. 2016 Mar 18;15:25. doi: 10.1186/s12943-016-0508-4 (PMC4797332; doi:10.1186/s12943-016-0508-4)
Supplement: Additional file 8: Table S1. — List of primers used for RT-PCR analysis. (PDF 40 kb) [file 12943_2016_508_MOESM8_ESM.pdf]

Table 1 Primers used in this study

Name	Sequence
GLIPR1-F	GACCCAGCACTAGCCCAAAT
GLIPR1-R	GTGATGGCGGAAGACACAGA
ErbB3-F	GGTGCTGGGCTTGCTTTT
ErbB3-R	CGTGGCTGGAGTTGGTGTTA
FGFR1-F	CAAACCAAACCGTATGCCCG
FGFR1-R:	AGGTGGCATAACGGACCTTG
FGFR2-F:	CGCTGGGGAATATACGTGCT
FGFR2-R:	AGTCTGGCTTCTTGGTCGTG
FGFR3-F	AACAAGTTTGGCAGCATCCG
FGFR3-R	TCCTTGTCGGTGGTGTTAGC
FGFR4-F	GGAAGGCAGTTGGTGGGAAG
FGFR4-R	GCTACTGTCAGCTCCTGCTC
